# The Physiological Therapeutics of Dypsomania

**Published:** 1901-02

**Authors:** C. A. F. Lindorme

**Affiliations:** Chicago, Ill.


### THE PHYSIOLOGICAL THERAPEUTICS OF DYPSOMANIA.

By C. A. F. LINDORME, Ph.D., M.D.,
Chicago, Ill.

The drink theory has changed considerably in the latter decades. Whether gold had anything to do with it or not, it has been claimed that there is none administered in the Keely-cure; or whether apocynium, as by some is asserted, was the working remedy. It is a fact that professionally not only, but popularly, the belief in physical remedying has got the better of the devil who formerly reigned tyranically in the matter. Conversely the pathology of dipsomania has been construed correspondingly, it has been declared a disease with no moral disparagement being unconditionally present.

*Prima facia* this is very captivating. The principle must hold that nothing physical can be moved metaphysically. But if the American-saloon keeper is anxious to have his "family entrance," or special ingress arrangement for female patrons, while in Spain, I myself have seen on the pier a lot of women standing around an English sailor lying dead drunk in a ditch, and crying for help, as though there were a person dying. As a fact they did not know such a thing as drunkenness. It is not well possible to explain this by exclusively physical causes. According to the descriptive Homer, the satirical Juvenal, the ancient Greeks and Romans knew a good deal on this line, and even if we are governed by climatic considerations, we cannot therewith explain the differences or modalities in the drink-habit, which appear, nationally, between French and German, compared with English and Americans. When the German goes to his "Setdel" or "Schoppen," his beer or his "Heurigee," he is "on duty;" he is as religious about it as going to church. If the stuff is bad he will kick, but he will go for all that. If an autochthon of Munich should not be able to be present, when the first "Bock" is being tapped, he would not know how to balance that in his curriculum. There is in the German drinking habit a sociable element which, in its anatomy, comprehends the very table the habitués sit down at, something which is not likewise found among the typical French. Whether a Frenchman goes to his absinthe or engages in a big spree (*faire la noce*) he is most particular about the strictly individual satisfaction he may derive from the arrangement; he is the man of the *petits verres;* although he is far from avoiding intoxication, he will in his scrupulousness of itemizing the *menu* exhibit the confidence that he will not get *soul,* and consider this an unfavorable return of his investment. The American, on the contrary, indulging in liquor, looks as though he were in a particular hurry to get drunk; he seems to be more or less aware that there is at the bottom no enjoyment in the business. The discomfort of the barroom, even, seems to be an injunction, "get your drink and be gone!" But it is a part of the program of life. He "does" this as he "does" a city in traveling. He has not yet got so far as to work out his own philosophy of life, and so he keeps awhile busy with overdoing European ways, just to make manifest what rotten principles they are which it has been our lot to inherit. John Bull, sitting down to his roast beef and beer, or steak and port, and keeping on drenching himself with the liquid after getting to the limit of stuffing himself with the solid, is bloated with the conviction that he fulfils an historical mission. If he cannot prove it by rote, in upholding the dearest of his dreams, that of having "come over with the Conqueror," he will do thus much for his historical mission, that he drinks as much as they did then, and so, rule Britannia. If a Boer war cannot be had every day, well, some other drunken brawl, they are always accommodated by their "Christian government."

As to the prognosis, we believe it is most favorable with the American, that is to say, the American with a will. The pseudo-Americans—those who, although divorced from the old hailing—continue to flirt with it, call themselves Germans, untrue to the new home, offer a resistance which, to the leaders of the prohibition movement, is a considerable puzzle. The genuine American inebriate, far from having made up his mind for good as to the question whether the drinking habit is an integrant part of the customary society, he is open to a contrary argument. But with the German, the drinking habit forms a part of his social philosophy. With the American, drinking is a filling up with beer or whiskey. With the German, it is an ethnological feature which forms the condition of the enhanced plane of homebred sociability; it stands nearer to his heart than the American citizenship, and an abolishment would carry with it an obliteration of the peculiar charm of the cherished German "Gemuethlichkeit." The homespun beer-philosopher of genuine Teutonic breed will admit with a smile, that intoxication is bad. But he will lay the fault at the door of nature, not his own, and at any rate uphold, that to stint himself with beer, or to say stop, so long as there is anybody who wants a comrade to realize with him the inborn spirit of German sociability, is a heap worse than the fumes which arise from too much and which must be borne as other strangulations of the pneumogastric. And this is not, perhaps, the tenet only of an inferior class of society. There was no more passionate believer in this sort of sport than prince Bismarck. Nay, more than that, art and intellectuality have combined to raise, by superior diction, by the invention of a distinguishing rite, by the finest in its form, noblest poetry and the most bewitching and inspiring music the beer-custom or wine-devotion as the case may be. So that the disciple, so far from feeling lowered, socially, if he finds himself a university student, in a soft summer night in the ditch with a fellow-student, keeps the scenery in jovially hugged remembrance among the wreaths of friendship which he holds dearest among his relics of the "happy days."

As particularly characteristic I give hereunder a translation of a celebrated ditty by v. Muehler, D.D., in his time member of the Royal Prussian cabinet, secretary of public worship, education and medical matters:

Right from the tavern do I arouse.
Now, street, how curious looks every house,
Both right and left turn'd reciprocated,
I trow, thou art, street, intoxicated.

And now, you lamp posts, what do I see ?
It seems you all have been on a spree;
You swagger and reel on the left and the right,
You are, I am confident, terribly tight.

And Luna, my girl, oh what a lark!
One eye is bright, and the other dark;
Tipsy thou must be, now that is clear,
Fie, maiden, fie, on thy drunken career.

All in a storm, the big and the small,
May venture in, I sober withal ?
I am scrupulous, 'tis a risky affair;
I'd rather again to the tavern repair.

Yet, as to analytical psychology, it would be a serious mistake to presume an intellectual authorship of poetry with regard to the drinking habit; the latter is ever so many centuries older than the jocose poetry of carousing ditties. We have a record of it nearly two thousand years old, by Tacitus, who mentions it as a time honored affair. The psychological order is, that it was not the poetry which provoked the drinking habit, but the drinking habit which gave occasion to the carol. This is wonderfully illustrated by Washington Irving in his adventures of Captain Bonneville. A company of trappers, or squatters, experienced the dire calamity to be out of alcohol. It was at some spot and period when and where they were used to carouse. It happened to be at Beer Springs, a place which was called so on account of the color of the water bubbling forth from the Rockies, resembling that of beer. The camp wanted a frolic and, nothing else being at hand, the water was drunk, and it served the purpose as well; the mirth was as great, and the only difference from other frolics was, that on the next morning there was nobody suffering from headache and indigestion.

I defy any psychologist to demonstrate passionate emotion as the result of mere nerve function. The object of the sensory impressions is intellection, understanding, and the very nature of this its indispensable requisite, especially in the reflective exhibition in the upper nerve-centres, is inhibition, delay, reiteration, inattentive pondering, which precludes passionate impetus, emotion. The motor nerves cannot functionate at all except with and by the blood-current. Nor can they functionate except according to the blood-current which they meet; hence the estimation of a thing according to the pedestal on which it is put:

> "The nightingale, if she should sing by day,
> When every goose is cackling, would be thought
> No better musician than the wren.
> How many things by season seasoned are
> To their right praise and true perfection."—*Shakespeare*

On the other hand there can be considerable influence of impressions, as conveyed through the nerves, if the nerve-cell, in its blood-intersection in the capillaries, meets a favorable mood. This is what is understood by prejudice, preconception; it exists in its foundation independent of all nerve-supply or connection, as the primitive exhibition of mentality in the positive utterance of the will it determines the conception which takes place on the basis, apparently, of the date of observation. It needs not be emphasized, therefore, that poetry like that of Dr. v. Muehler, D.D., is bound to work mischief among the youth of his country, especially with the additional charm that is given it by an extremely fit melody; all this substantiates in the mind of the people a fictitious reality, creating an environment which must lower upon the character a cloud of untruth and equivocation.

Physically there is no charm in the alcoholic seasoning. Oft and anon one meets with individuals who claimed that they liked whiskey and tobacco from the first moment that, as boys, they got any. But there are people with very imaginative memory, and as a rule we dare say that the taste of a child rejects vigorously the one as well as the other. I had a friend, I wish I had never met him, who used to say, when seventeen years old, "I wish I could live in a state of perpetual drunkenness." According to the common, transcendental, metaphysical way of interpreting or diagnosing such cases, we should have to classify that as hypersensuality. But in this case it was not present. There was no hypersensuality in that lad. He was an exceedingly bright, but wildly eccentric fellow. Before girls, however, he was a lame duck. I am of the firm conviction that what he ailed was lack of energy from *want* of sensualty. He was an extraordinarily good scholar. But his ambition did not tend beyond the mediocre; poor sport and small game; and he was aware of it himself, although he heeled it behind an eccentric exhibit. That is why he longed for the drowsiness of the narcotization; feeling himself not enough man to carve out in reality an ideal that could gratify him, he took his refuge in the obfuscation of his brain. The personality he built up was corrupt. He mistook extravagance for eminence. After he had settled in his liquor habit thoroughly in London, England, he went to Havana and died there a week after of yellow fever. When he was warned that the climate did not tolerate such indulgence, he answered, "O, that does not hurt me." Thus the characteristic sign of extravagant self-feeling did not fail to indicate the seat of the trouble.

Inebriates are sometimes made in a very off-hand way. I had myself the misfortune of such an experience. When I was about eleven years old, I was taken with my brother on a trip my father made with my mother to Hameln, or Hamelin, as it is spelled here, the place of which the curious story is abroad of the piper, the rats and the children, which then was more celebrated for its beer. The rocky vaults were on a high hill from which one had a wonderful view into the Weser valley of the romantic Westphalia, so poetically sung by Freiligrath, and we went, as a matter of course, to see the sight, in doing which the beer was not forgotten. I had to taste it too, and since at that time I could not follow the advice of an Italian writer, in a sweet little story, "Il Signior Io," (Mr. I myself), never to take either German beer or German metaphysics but in big gulps, I proceeded with the natural cautiousness before a strange affair, and brought the equivocal stuff under the most unfavorable circumstances in contact with my tongue and palate. I withdrew the mug, and made a wry face. It tasted horrid, compared with the milk, chocolate and summer drinks my good mother made at home, and my critique was read from my features. But the ingenious exhibit of my sentiment was not countenanced as I expected; it met with a peal of laughter by all the persons around, and scared by this unanimous manifestation of public opin on, I resumed my trial, and came out second best this time.

I am not prepared to utter an opinion on the question whether there is an hereditary taint of dipsomania. But I am inclined to take the word of our great medical practitioners in that line for it. On the other hand I would venture the supposition that there is more metaphysical influence by bad example and adulterated public opinion than is dwelt upon by our experts like Crothers. It is not anything directly attractive, but indirect suggestion through ambition and lack of occupation for the ten fingers. One needs only to watch in the streets the boys lighting their cigarettes, to become imbued with the enormous earnestness they attach to this act of their growing manhood, deeply concerning their whole time! And how a group is carried by the most obstreperous character in it! There is nothing to bolster the shy, intimidated common sense. Eight years after I had in Hamlin so heroically made good on the spot my lack of temerity, I laid a wager to drink twelve tumblers full of beer before my adversary could eat a twenty-four-hours old roll, and, *horribile dictu* (horrid to tell), I won it, too.

If Portia, in order to avoid being obliged to listen to the wooing of the Duke of Saxony's nephew, a "sponge," in case he should come out best man in the game with the caskets, directs her maid, "Therefore, for fear of the worst, I pray thee, set a deep glass of Rheinish wine on the contrary casket, for, if the devil be within and that temptation without, I know he will choose it."—(Shakespeare.) There is in this concatenation all the coercive of it, not a physical compulsion, originally, but a metaphysical one. A child experiences nothing attractive in Rhenish wine. They keep in the "Rathhauskeller" (Municipal vaults) in Bremen (Germany) "Rose" and "Zwoelf Apostel" wine, about three hundred years old and priced highest, which, barring its flavor, is the most detestable stuff which can be imagined. A baby would estimate it no higher than vinegar. It is no better. All its merit is in its great age, and this notoriously spoils Rhenish wine. But public opinion has settled the matter, and, like in fashion, one foolishness draws the other. If that Saxon "Gauch," when young and all the time, had not heard that it is manly to drink "deep" bumpers; if, from infancy up, he had not seen that it was a chivalrous act to adore the fair sex by drinking one's self under the table, they would never have fancied it, and this goes far to show where is the guilt in the matter.

The human will is a very peculiar agency, a curious faculty, indeed. It is understood, theoretically, by most men, and frequently, we dare say, misunderstood by the man who has it. Everybody thinks he can handle his will according to—his will. But, the meeting breaking up, nobody knows who is who. The routine interpretation is, that the will is made up in the encephalon, according to the nerve impressions. But if this were so, the will ought to be the same when the nerve-impressions are the same. Accordingly the stratagem of Portia ought to work uniformly, which it is far from doing. There is a subjective factor, and this is the red blood corpuscles, as the only bodies the concomitancy of which is indispensable to a nerve-action. Hence a peculiar independence of the will. The red blood corpuscles floating freely in the serum, the data of observation in the brain cannot determine the drift of action in man, except in agreement with his inner disposition. There is a latitude of option, and consequently in man and his motivation there is a different aspect of cause and effect than in the merely physical world. In most cases, on account of the secrecy of this relation, unless it is a very common one, it seems exorbitant and out of proportion. This substantiates the dramatic and histrionic art. A mighty knight had an angry feud with a crafty and personally titanic neighbor, whom he tried in vain to subdue. He promised a high reward to any one who would bring him in, fettered; and one fine morning, who came in but the tender daughter of the grim tyrant, with the wanted knight, fettered with a rose silken ribbon, the athletic warrior in steel following the damsel like a lamb in leish, asking the gentleman for her household. Well, daddy wanted first to be cross, but soon understood it would be more profitable to have that ambulating steel-ramrod as a son-in-law than an adversary, and she got him—(Fliegende Blaetter). Hence the consistency of coercion, as in cause and effect, and the personal liberty of the individual. If it is said of Napoleon that he was compelled to go to Russia, it may be said also that the giant knight was more effectually fettered with the rose-silken ribbon than with a cord, but it was in both cases because they wanted to. Was there anybody to compel Napoleon going to Russia? There was nothing anybody was so little allowed to have a will in the matter as that war, and he consequently put in more of his own personality than anything else, and to deny his having had a will in it is linguistic vagary.

The will, we have said, as a faculty, is represented by the red blood-corpuscles. This is correct. But the will cannot exhibit except in taking a special direction, or with reference to some special thing. It cannot become known, therefore, except in manifesting itself in the objects presented to it by the nervous apparatus. Hence the appearance of originality in the latter. This is an illusion. In the nervous system the will becomes apparent. Its original plant is in the red blood-corpuscles. If this was not so, then the nerve-impressions ought to work the same way, lead in all persons to the same results, something which notoriously they do not do, the subjectivity of the red blood-disks playing its part always, turning thereby the relation of cause and effect in the unorganized nature into motivation and deed in man. If this was not so, psychology would be like chopping wood.

And the reader must not overlook a circumstance which is leading in this direction. The material offered by the nerve-impressions to the will of man is very chaotic, fragmentary, for the matter of that; it is far from being presented to the will in the order in which it is realized here. The nerve-cells receive the single data of observation as conducted through the nerves. That is all. It remains to the red blood disks to compare, to sift, to conclude, and in so doing nothing is more natural than that a good deal of coloring and cooking goes on at the same time, the will yielding to the temptation of doing the supplementary work left to it, to put the data in shape contained in the brain, so as to suit itself. There will be more or less temptation according to the person. But there is no man in whom the sovereign option in the interpretation of the data of the brain, to please itself, will not to some extent connive with some hidden fancy, and it is the influence of this kind of cherished individualism which keeps up habits and fosters vices among men not a peculiar disorderly turn of the intellection in the brain.

As to hypersensuality this is simply a metaphysical slander. It dates from the times of mediæval vocabulation, when ethics were not thought possible except in transcendental evanescence. Since we have learned to understand nature in perfect unity of thought, we need not be afraid of hypersensuality, because it carries its share of idealism with it, to make up nature in its orderly and beneficent complete composition. Moreover, as in the case of my eccentric friend, a better understanding of the relevant facts speaks against such supposition of hypersensuality. Statistics for instance prove that the heaviest percentage of cases of morphine-addiction falls on unfortunate brethren-practitioners. Now, is that owing to an extra degree of hypersensuality met among physicsans? It would be ridiculous to suppose such a thing. It is the outworking of the ambition which is at the bottom of so much mischief. Man wants to run not only the affairs of man, of society, but of nature as such. Hence the ready handling of the perniciously handy pop-gun. And this especially in the young physician who has his hands idle, and only too often his brain too. He wants to do something. Proper work not being on hand, it is the improper work he takes in hand, and his own body is used as experiment-station. But here, so far from succumbing to a plus of sensuality, it is the traditional metaphysical abstraction-faultiness he falls a victim to. Nature is considered under the aspect of pathology, merely, and interpreting her phases, in the narrow-mindedness of old transcendentality, as the utterance of step-motherly antipathy, her supposed violence is in therapeutics met by adequately destructive agencies, and "the battle is lost and won," usually with the result as in the case of the magician-apprentice (Goethe's Faust) who had learned of his master to set the charmed besom a-sweeping, but could not restore its quiescence, so that he, with all the rest, was swept out of the shop. Now, with more sensuality, that means with more manliness, the medical adept would have had more confidence in nature, and following physiology, would have found in it the reconstructive means of recovery.

Crothers and his school are certainly right that dipsomania is a disease. But the most effective therapeutics is in a rooting up of the crude prejudice that the "strongest" wine is port with thirty odd per cent. of alcohol in it. If it is a fact, even, for which recently Dr. Thompson, as true, declared it honest to stand out, that alcohol is a respiratory nutriment, it is no less authentic that such nutriment can be administered in surpassingly more wholesome form. Honey is ever so much more wholesome than alcohol.

Viewing our subject from a scientific or an empirical standpoint, it is certain that vice is a bad habit, and can in no way better be mended but by setting up in place a good habit. "Rien n'eloigne le mal comme le bien." (Helvetius.) Nothing removes the bad so well as the good. Forty-five years after my unfortunate incipient issue in Hameln, I discovered that I got sick by indulging in liquor, beer and cigars. I was almost choked by accumulation of phlegm. Now, then, this recognition once fairly established, it took me just five minutes to become a tee-totaler, not a pledged but an upright one. I turned vegetarian at the same time, and have continued to be one since, and this was not owing to any superior intellectuality but merely to a determinate will, the will to judge for myself. This will there was already in Hameln. But in a most damnable way it was adulterated by cross-purposes, taking the edge away from my character and making a play-toy out of me in the hands of the most brainless fop. It was a *leze-majeste* of the divine nature of man, a crime against my personality, the budding personality in my boyhood. With most wonderful gifts nature had bountifully provided me, to meet the world on even footing, and in abominable perversion the most valuable and salient qualities were corrupted to the counterfeit of a caricature. I was flung into the contest of life with all the odds against me, and nothing short of a gigantic struggle could revindicate my character from the thraldom of the old rut, establishing the independence of my will again opposite time-worn ancester-doctrine.

Was there hypersensuality? Yes, there was. But this was just what saved me. By their hobgoblin-abstraction-bamboozle they had gotten a good deal of backbone-straightforwardness out of me. But there was left a stock of genuine Westfalian stiff-neckedness which asserted itself against their mendacious civilization-tomfoolery, and which they could not overcome and disinfranchise.

The empty silliness of transcendental metaphysics has life turned away from itself, and it is high time to return to scientific principles of physiology: Is not eating and drinking the A B C of life? Should not, therefore, the purity and sensibility or the verity of this A B C be the gospel of education? As it is, Eating as well as Drinking, nay, Smelling, Hearing and Touching is an indignity. Not only in boarding-houses but in hospitals I have seen the inmates in insane uniformity grasp the salt-cellar and the pepper box, and with might and main shake them over the dishes which had been brought them, and, mind well, without first trying or tasting them. That was a seasoning three times over. First and probably to all intent and purpose sensibly adequately, nature seasoned the aliment by providing it with the necessary salts, then the cook did it, *in dubio* copiously; lastly, the consumer did it lavishly.

As a fact, man does not know what he wants, and most times does not do what he really likes, so that in every phase of his life we hear a lament about the wrong management of the preceding one. And that the ancient Greeks in the same way used to get on the wrong side of the fence indicates their emphasizing in philosophy the *gnothi seauton*, know thyself! But if it seems, no philosophical system affording the means of solving the problem, that it is insoluble, it should be borne in mind that a fair instruction and in good sooth methodologically, an education, was never undertaken; the metaphysical slander of sensuality is so heinously thorough that it blights the most tender blossoms of human life, infesting it with the abstraction-plague. The other day a kindergarten journal fell into my hands, and what was the main contents? A long wail that the girl teachers do not know what to do with their pledges. Now, can there be a more pregnant expression in the confession of intellectual pauperism than this statement? There is the appaling statistics of increase of the terrible trinity of drunkenness, crime and insanity, these fiendish obstacles of progress and humanity, and opposite this exhibit of degredation of grown man, the most elementary teachers tell us they do not know what to do to preserve the innocence delivered up to their care!

The fault, however, lies again at the door of transcendental metaphysics. In all other lines of work, if there is any thorough mending to be done, it is extended to all its detail. That is much too punctilious for metaphysics; it proceeds summarily; it gets its henchmen, the law, to put it in terse language, and says to the individual: "Look here, you please be good, else you will see how bad I am."

Physiology does not proceed in such a style. If there is mending to be done, she looks to all the detail, the minutest, even, of the body to be mended, and then applies the proper remedies. Now, the case in hand is this: The personality of the grown people is more or less in a process of corruption, caused either by specific vices or generally by an inordinate life, and since the personality of man is constituted by the relation of the will in the red-bloodcorpuscles, to the data of observation in the nerve-cells, proportionately to each other, attention must be given on the one hand to the functionating of blood-making organs, the alimentary canal, the liver, the pancreas, the spleen, the bones, and, not to forget the skin, as the finishing, as it were, of the body; and, on the other hand, to that of the five senses, which is going on in the most intimate meeting-place of blood and nerves, the brain.

Now, naturally the *pepiniere* for the said work should be the homes, the cradle of adult life. But of all ignorance in families, hardly any being more monstrous than that of child-training, they are out of the question, and so, as the next best, the kindergarten comes to the fore, not that there may be any virtue in the said establishments, but because they can easiest, under medical supervision at least, be made available for the purpose. The first thing to do, as a matter of course, will be the training of the girl-teachers. The senses of the same are more neglected, perhaps, than those of the infants, and they will have as hard a work to catch up as the backwoodsmen had when they were confronted with the red man in the time of the country's pioneer work. But then a field opens up before their diligence which is vaster but infinitely more grateful than any other one in the whole domain of pedagogic application. Then it will never occur again in the kindergarten literature such an indignity as the wail that they do not know what to do with their precious pledges.

